# Effects of Sex, Training, and Maturity Status on the Cardiopulmonary and Muscle Deoxygenation Responses during Incremental Ramp Exercise

**DOI:** 10.3390/ijerph19127410

**Published:** 2022-06-16

**Authors:** Adam Runacres, Kelly Mackintosh, Tim Evans, Melitta A. McNarry

**Affiliations:** 1 Applied Sports Technology Exercise and Medicine Research Centre (A-STEM), Swansea University, Swansea SA1 8EN, UK; a.runacres@mmu.ac.uk (A.R.); k.mackintosh@swansea.ac.uk (K.M.); 2 Sport Wales, Sofia Gardens, Cardiff CF11 9XR, UK; tim.evans@sport.wales

**Keywords:** aerobic fitness, children, exercise, performance, physiology

## Abstract

Whilst participation in regular exercise and sport has generally increased over recent decades globally, fundamental questions remain regarding the influence of growth, maturation, and sex on the magnitude of training response throughout adolescence. Trained (108 participants, 43 girls; age: 14.3 ± 1.8 years) and untrained (108 participants, 43 girls; age: 14.7 ± 1.7 years) adolescents completed an incremental ramp test to exhaustion during which breath by gas exchange, beat-by-beat heart rate (HR), stroke volume (SV) and cardiac output ($\overset{\cdot }{Q}$) and muscle deoxygenation were assessed. Device-based physical activity was also assessed over seven consecutive days. Boys, irrespective of training status, had a significantly higher absolute (2.65 ± 0.70 L min^−1^ vs. 2.01 ± 0.45 L min^−1^, *p* < 0.01) and allometrically scaled (183.8 ± 31.4 mL·kg^−b^ min^−1^ vs. 146.5 ± 28.5 mL·kg^−b^ min^−1^, *p* < 0.01) peak oxygen uptake ($\overset{\cdot }{V}O_{2}$) than girls. There were no sex differences in peak HR, SV or $\overset{\cdot }{Q}$ but boys had a higher muscle deoxygenation plateau when expressed against absolute work rate and $\overset{\cdot }{V}O_{2}$ (*p* < 0.05). Muscle deoxygenation appears to be more important in determining the sex differences in peak $\overset{\cdot }{V}O_{2}$ in youth. Future research should examine the effects of sex on the response to different training methodologies in youth.

## 1. Introduction

Prior to COVID-19, 48% of children and adolescents in Wales participated in extra-curricular sports three or more times a week, an increase of 4% compared to 2015 [1]. This upward trend in sports participation is encouraging given the health benefits associated with exercise during childhood and adolescence [2,3]. However, despite this widespread participation in intensive training, fundamental questions remain regarding the short-term physiological responses to training in youth [4]. Indeed, one area that has received renewed interest over the last decade is the concept of a maturational threshold which suggests that pubertal children may experience an accelerated adaptation to training stimuli relative to their pre-pubertal counterparts, mediated by increases in circulating androgenic hormones [5]. The existence of a maturational threshold remains highly debated [6,7], with suggestions that it may be dependent on the specific parameter in question [8] or, possibly, sex [9].

Despite the research and practical interest in the influence of maturity on the training responses in youth, few studies have considered the interaction of training with the concomitant effects of growth and maturation according to sex [4,6,9]. Puberty is highly sexually dimorphic, with significant differences in the timing and tempo of maturity-onset and hormonal milieus [10,11]. Whilst no studies have specifically sought to compare the influence of training in boys and girls, marked differences are apparent in the literature suggesting that, in contrast to boys [12,13], training was not associated with significant gains in pre-pubertal girls [14].

More recent studies have suggested these findings are more likely to reflect methodological factors rather than a physiological inability to respond to training [9]. Indeed, when peak $\overset{\cdot }{V}O_{2}$ is rigorously determined, studies report that girls experience a similar degree of trainability to their male counterparts [9,11,15]. Specifically, McNarry et al. [15] found trained pre-pubertal girls to have a 17.5% greater peak $\overset{\cdot }{V}O_{2}$ than their untrained counterparts, with similar training differences in absolute peak $\overset{\cdot }{V}O_{2}$ in pubertal (21.5%) and post-pubertal (17.5%) adolescents. These observed improvements were proposed to be mediated by an increased gas exchange threshold (GET), an absence of a plateau in the stroke volume (SV) response and a rightward shift of the deoxygenated haemoglobin ([HHb]) response during incremental ramp exercise [15]. Whether similar mechanisms are responsible for the training-related increases in peak $\overset{\cdot }{V}O_{2}$ reported in boys largely remains to be established [8,9]. Whilst morphological and functional myocardial adaptations were reported in boys [16,17], no studies have investigated the influence of training on peripheral oxygen extraction during an incremental ramp exercise.

Interestingly, the few studies that have investigated the effect of sex on the development of peak $\overset{\cdot }{V}O_{2}$ suggest that sexual dimorphism is evident even in pre-pubertal children [18,19]. Specifically, when peak $\overset{\cdot }{V}O_{2}$ is normalised for body mass, pre-pubertal boys have a 10–15% greater peak $\overset{\cdot }{V}O_{2}$ than girls [18], which may be attributable to a higher oxygen delivery capacity mediated by a greater maximal stroke volume (SV_max_) and, consequently, cardiac output ($\overset{\cdot }{Q}$_max_). However, one of the key considerations when comparing boys and girls is the differing body compositions, with girls having a higher percentage of body fat than boys from ~10 years of age [20], which questions the utility of ratio scaling by body mass [21]. Yet, even when pre-pubertal boys and girls are matched for lean body mass, boys still demonstrate a ~15% higher peak $\overset{\cdot }{V}O_{2}$ [18], despite no sex differences in SV_max_, $\overset{\cdot }{Q}$_max_, or the structural properties of the myocardium [17]. This contradicts previous suggestions that the sex differences may be related to differences in oxygen delivery. In contrast, Winsley et al. [18] suggested that these sex differences may be attributable to sex differences in the ability to extract O_2_ from the working muscles. More specifically, a greater maximal arteriovenous difference (a-$\bar{v}$O_2diff_), an indicator of peripheral oxygen extraction, was observed in boys. However, the a-$\bar{v}$O_2diff_ only offers a macroscopic overview of peripheral oxygen extraction in contrast to the microvasculature insights afforded by near-infrared spectroscopy (NIRS; [18,22]). Nonetheless, these findings were corroborated by McNarry et al. [23] who reported that the plateau of the [HHb] response during a ramp exercise explained ~12% of the variance in peak $\overset{\cdot }{V}O_{2}$ between sexes after accounting for fat-free mass (FFM), the GET and body fatness. However, it is pertinent to note that most of the participants in both studies were pre-pubertal, precluding inferences as to the relative contribution of the oxygen delivery and extraction in pubertal and post-pubertal adolescents. Therefore, further research is warranted to ascertain whether similar mechanisms underpin potential sex differences in adolescents.

The aim of this study was to investigate the influence of training on the aerobic fitness of youth and whether this or the mechanisms underpinning it differ according to sex. It was hypothesised that differences in oxygen extraction would, at least in part, explain the sexual dimorphism in peak $\overset{\cdot }{V}O_{2}$ in children and adolescents. 

## 2. Materials and Methods

### 2.1. Participant Characteristics

Written parent/guardian consent and participant assent were obtained, and a pre-screening medical questionnaire completed prior to any testing. Participants were excluded if they had any known pre-existing condition that would prevent them from completing all experimental procedures. 

Trained children and adolescents were recruited through the respective sport’s National Governing Body (NGB), with all trained children and adolescents part of a long-term athlete development programme. All players, across all age groups, were invited to take part as part of the yearly registration with the NGB. Trained participants had completed an average of 10 ± 5 h∙week^−1^ of training for at least two years prior to study entry. A typical training schedule included sport-specific drills, tactical awareness sessions, and usually finished with a high-intensity small-sided game or match play scenario. Untrained participants were recruited from local schools across South Wales and were not involved in any extra-curricular sports-based activity. Participants were excluded if they failed to complete all exercise elements of the study, or had any known cardiovascular, metabolic, or chronic condition. The final sample consisted of 216 participants, of which 108 were trained (43 girls; age: 14.3 ± 1.8 years) and 108 were untrained (43 girls; age: 14.7 ± 1.7 years). The participants’ characteristics and physical activity levels are presented in Table 1. 

### 2.2. Experimental Procedures

On arrival at the laboratory, blood pressure was recorded after five minutes of rest using an automated blood pressure monitor (Omron MX3, Milton Keynes, UK). Stature and sitting stature were then measured to the nearest 0.1 cm using a Holtain Stadiometer (Holtain, Crymych, Dyfed, UK), and body mass recorded to the nearest 0.1 kg using electronic scales (Seca 803, Seca, Chino, CA, USA). Maturity status was estimated using the equations of Mirwald et al. [24], with participants ≥1 year from, between −0.99 and 0.99 years from, and ≥1 years post peak height velocity classified as pre-, circa- and post-pubertal, respectively. 

Peak $\overset{\cdot }{V}O_{2}$ was assessed using an incremental ramp test to volitional exhaustion on a cycle ergometer (Lode Excalibur Sport, Groningen, Netherlands). Specifically, following a three-minute warm-up at 10 W, the resistance increased by 20–25 W·min^−1^, depending on the participant’s age. All participants were instructed to maintain a cadence of 60–80 revolutions per minute (rpm) throughout the test, with the inability to maintain a cadence above 50 rpm despite strong verbal encouragement defined as volitional exhaustion. Inspired and expired air were collected on a breath-by-breath basis using a Vyntus Metabolic Cart (VYAIRE medical Ltd., Mettawa, IL, USA), with beat-by-beat heart rate (HR), stroke volume (SV) and estimated cardiac output ($\overset{\cdot }{Q}$) assessed using a thoracic bioelectrical impedance device (Physioflow, Paris, France) with the six electrodes placed according to the recommendations of Welsman et al. [25]. Finally, muscle deoxygenation was assessed throughout the exercise protocol using a portable NIRS device (PortaMon, Artinis Medical Systems, Einsteinweg, Netherlands), placed on the m. vastus lateralis of the dominant leg [22,23]. The NIRS device was secured to the m. vastus lateralis using sports tape, with blackout cloths also used to prevent ambient light from distorting the NIRS signal. The NIRS device was zeroed whilst the participant was seated and relaxed on the cycle ergometer, prior to data acquisition. The Vyntus, PhysioFlow and Portamon were all calibrated in line with manufacturer instructions prior to each peak $\overset{\cdot }{V}O_{2}$ test, and other studies of this type [7,22].

To verify a maximal effort during the incremental ramp test, all participants completed a supramaximal validation bout after 15 min of rest. This bout involved participants completing three minutes at 10 W before undergoing a near-instantaneous transition to 105% of the peak power achieved during the incremental ramp test. Participants were instructed to maintain a cadence >50 rpm for as long as possible, with gas exchange continuously measured. For the subsequent seven days, participant’s habitual physical activity was assessed at 100 Hz using a GT3X accelerometer (ActiGraph, Pensacola, FL, USA) worn on the right hip. All participants were instructed to wear the accelerometer 24 h a day except for prolonged water-based activities. Participants were also asked to complete a sleep log detailing periods of monitor removal, waking time, and bedtime. 

### 2.3. Data Processing

The raw $\overset{\cdot }{V}O_{2}$ data from the incremental ramp test and supramaximal validation bout were averaged into 10 s bins, with peak $\overset{\cdot }{V}O_{2}$ defined as the highest 10 s moving average during the incremental ramp test. To aid comparisons according to sex, maturity, and training status, peak $\overset{\cdot }{V}O_{2}$ was allometrically scaled by body mass, using methods detailed elsewhere [26]. The GET was determined using the V-slope method [27] and defined as the point at which carbon dioxide output ($\overset{\cdot }{V}CO_{2}$) rose disproportionally to $\overset{\cdot }{V}O_{2}$. The GET was expressed in absolute terms (L min^−1^) and as a percentage of peak $\overset{\cdot }{V}O_{2}$. The kinetics of the initial $\overset{\cdot }{V}O_{2}$ response were quantified using the mean response time (MRT), determined from the onset of the ramp forcing function to the intersection point of the baseline $\overset{\cdot }{V}O_{2}$ and a backward extrapolation of the slope of $\overset{\cdot }{V}O_{2}$ as a function of time [28]. Finally, the O_2_ cost of exercise was quantified according to the gain, calculated as the average change in $\overset{\cdot }{V}O_{2}$ per W over the entire ramp test [28]. 

The data obtained from the PhysioFlow were averaged into 15 s bins and maximum heart rate (HR_max_), stroke volume (SV_max_) and cardiac output ($\overset{\cdot }{Q}$_max_) defined as the highest 15 s moving average. All cardiac measures (HR_max_, SV_max_, and $\overset{\cdot }{Q}$_max_) occurred at the same time point for all participants. SV_max_ and $\overset{\cdot }{Q}$_max_ were subsequently allometrically scaled to body surface area (BSA), estimated according to the predictive equations of Haycock et al. [29]. Additionally, to estimate the balance between O_2_ delivery and extraction, the peak a-$\bar{v}$O_2diff_ was calculated by rearrangement of the Fick equation Peak $\overset{\cdot }{V}O_{2}$/$\overset{\cdot }{Q}$_max_. Prior to analysis, the NIRS-derived [HHb] was averaged into 5 s bins, baseline corrected and normalised to end-exercise values. A sigmoidal function was then used to ascertain the relationship between [HHb] and peak $\overset{\cdot }{V}O_{2}$ and work rate in both absolute and relative terms [23]. 

The accelerometer data were downloaded into 15 s epochs using the ActiLife Software (v6.13.4.0, ActiGraph, Pensacola, FL, USA) allowing the Evenson et al. [30] cut-points to be applied which were demonstrated to accurately quantify sedentary time (SED) and moderate-to-vigorous physical activities (MVPA; [31]). Wear-time was set to >8 h·day^−1^ on any three days, shown to provide an accurate and reliable estimation of children’s physical activity [32].

### 2.4. Statistical Analysis

All statistical analyses were conducted in SPSS (version 26, IBM, Portsmouth, UK), with values presented as mean ± SD. An ANCOVA, covarying for maturity status, was used to establish the effect of sex and training status and any interactions. Cohens *d* was also calculated, with ≤0.20, ≥0.21–≤0.60, ≥0.61–≤0.80, and ≥0.81 considered a trivial, moderate, large and very large effect, respectively [13]. 

## 3. Results

There were no significant sex- or training-related differences in any anthropometric variable when accounting for maturity (Table 1). However, boys engaged in significantly more MVPA than girls, irrespective of training status (*F*_(1,215)_ = 6.8, *p* > 0.05, *d* = 0.23). 

### 3.1. Influence of Training

Trained participants had a higher peak $\overset{\cdot }{V}O_{2}$ than their untrained counterparts (*F*_(1,215)_ = 10.1, *p* < 0.01, *d* = 0.03), which persisted even after allometrically scaling peak $\overset{\cdot }{V}O_{2}$ for body mass (*F*_(1,215)_ = 11.7, *p* < 0.01, *d* = 0.16; Table 2). Furthermore, trained athletes had a higher maximal power (*F*_(1,215)_ = 19.9, *p* < 0.01, *d* = 0.04), SV_max_ (*F*_(1,215)_ = 2.1, *p* < 0.05, *d* = 0.01) and $\overset{\cdot }{Q}$_max_ (*F*_(1,215)_ = 2.1, *p* < 0.05, *d* = 0.03), but a slower MRT (*F*_(1,215)_ = 4.5, *p* < 0.01, *d* = 0.65), than their untrained counterparts. Contrastingly, there was no significant difference in the absolute or relative GET, gain, HR_max_, maximal a-$\bar{v}$O_2diff_ or allometrically scaled SV_max_ or $\overset{\cdot }{Q}$_max_ between training groups (all *p* > 0.05).

Trained youth had a less steep [HHb] slope when expressed against absolute $\overset{\cdot }{V}O_{2}$ (*F*_(1,215)_ = 8.6, *p* < 0.01, *d* = 0.28), however, when expressed against work rate (both absolute and relative) or relative $\overset{\cdot }{V}O_{2}$, no significant differences were evident (Table 3). Trained children and adolescents had a higher *c*/*d* and plateau than untrained children and adolescents, irrespective of whether [HHb] was expressed against absolute work rate (*p* < 0.01) or $\overset{\cdot }{V}O_{2}$ (*p* < 0.01). However, when [HHb] was expressed against relative $\overset{\cdot }{V}O_{2}$, these differences were ameliorated. Finally, as a function of relative work rate, only the plateau remained higher in the trained than untrained participants (*F*_(1,215)_ = 4.9, *p* < 0.01, *d* = 0.88). 

### 3.2. Influence of Sex

Boys had a higher absolute (*F*_(1,215)_ = 25.5, *p* < 0.01, *d* = 0.74) and allometrically scaled peak $\overset{\cdot }{V}O_{2}$ (*F*_(1,215)_ = 27.5, *p* < 0.01, *d* = 0.29) and absolute GET (*F*_(1,215)_ = 11.4, *p* < 0.01, *d* = 0.56; Table 2) than girls. However, when the GET was expressed relative to peak $\overset{\cdot }{V}O_{2}$, no sex difference was evident (*p* > 0.39). Boys were also characterised by a slower MRT (*F*_(1,215)_ = 8.7, *p* < 0.01, *d* = −0.53), a greater gain (*F*_(1,215)_ = 29.8, *p* < 0.01, *d* = 0.20), peak power (*F*_(1,215)_ = 13.1, *p* < 0.01, *d* = 0.32) and maximal a-$\bar{v}$O_2diff_ (*F*_(1,215)_ = 14.0, *p* < 0.01, *d* = 0.99). However, no sex differences were observed for either absolute or scaled $\overset{\cdot }{Q}$_max_ (*p* = 0.25), SV_max_ (*p* = 0.39), or HR_max_ (*p* = 0.24).

When [HHb] was expressed against absolute $\overset{\cdot }{V}O_{2}$, boys had a higher *c*/*d* (*F*_(1,215)_ = 19.1, *p* < +−0.05, *d* = 0.79) and plateau (*F*_(1,215)_ = 18.6, *p* < 0.05, *d* = 0.76). However, when [HHb] was a function of relative $\overset{\cdot }{V}O_{2}$, no sex differences were found (Table 3). Similarly, when [HHb] was expressed against absolute work rate, boys had a higher *c*/*d* (*F*_(1,215)_ = 8.2, *p* < 0.01, *d* = 0.59) and plateau (*F*_(1,215)_ = 4.0, *p* < 0.01, *d* = 0.48) compared to girls, but no differences were evident when [HHb] was expressed against relative work rate. There were no significant differences in the amplitude or slope of the [HHb] response expressed against any variable between boys and girls.

### 3.3. Interaction Effects

Significant sex and training interactions were observed for peak $\overset{\cdot }{V}O_{2}$ (*F*_(1,215)_ = 10.1, *p* < 0.01), SV_max_ (*F*_(1,215)_ = 4.9, *p* < 0.05) and $\overset{\cdot }{Q}$_max_ (*F*_(1,215)_ = 5.5, *p* < 0.05). Specifically, there was a greater difference between trained and untrained girls than boys for peak $\overset{\cdot }{V}O_{2}$ (8.5% vs. −0.8%, respectively), SV_max_ (12.3% vs. 8.4%, respectively) and $\overset{\cdot }{Q}$_max_ (30.2% vs. 15.5%, respectively). However, when allometrically scaled, none of these interaction effects persisted. An interaction effect was also evident for maximal power (*F*_(1,249)_ = 25.1, *p* < 0.01), with less difference between trained and untrained boys (12.6%) compared to trained and untrained girls (36.9%). A significant sex and training interaction was also evident in the [HHb] plateau when expressed against relative work rate, with a smaller difference between trained and untrained girls (41.3%) compared to boys (61.0%). Moreover, when [HHb] was expressed against absolute $\overset{\cdot }{V}O_{2}$, a greater difference was observed in the *c*/*d* (12.0% vs. 56.1%) and plateau (13.6% vs. 47.0%) of trained and untrained girls than boys, respectively. However, when [HHb] was expressed against relative $\overset{\cdot }{V}O_{2}$, a significant interaction was only found for the plateau (7.0% vs. 16.4%).

## 4. Discussion

The primary aim of this study was to explore the role of sex in determining the effect of training on pulmonary $\overset{\cdot }{V}O_{2}$, haemodynamic and oxygen extraction responses, accounting for maturity. The key finding was that the influence of training during youth was dependent on sex, with greater training-related differences in the peak $\overset{\cdot }{V}O_{2}$ of girls compared to their male counterparts. Despite this increased magnitude of training-related differences, boys still demonstrated a greater absolute, and scaled, peak $\overset{\cdot }{V}O_{2}$ than girls, irrespective of training status. The mechanisms underpinning this greater aerobic capacity in boys appear to be related to differences in oxygen extraction, with no differences in any of the haemodynamic (SV_max_ HR_max_ or $\overset{\cdot }{Q}$_max_) responses to exercise according to training status once normalised for body size. However, boys had a significantly higher [HHb] plateau when expressed against absolute $\overset{\cdot }{V}O_{2}$ and work rate, although these sex differences were ameliorated when expressed against relative $\overset{\cdot }{V}O_{2}$ and work rate. This, therefore, suggests that boys and girls have similar levels of oxygen extraction for the same relative sub-maximal work rate and $\overset{\cdot }{V}O_{2}$. 

These findings provide novel insights into the comparative trainability of youth according to sex and should be considered in long-term athlete development plans.

Training is associated with a significantly higher peak $\overset{\cdot }{V}O_{2}$ in youth, irrespective of maturity, and therefore represents a potent stimulus to aerobic fitness [6,7,8,9]. In the current study, a significant training and sex interaction was observed for peak $\overset{\cdot }{V}O_{2}$, suggesting girls may display a greater magnitude of change in response to a training stimulus compared to boys. This could be due to girls’ lower baseline fitness levels as higher initial levels are known to attenuate the response to training [33]. However, this seems unlikely to fully explain this interaction given the habitually trained nature of these participants. Alternatively, the significant difference in MVPA levels between boys and girls, irrespective of training status, may indicate a significant effect of physical activity on peak $\overset{\cdot }{V}O_{2}$ but the evidence surrounding habitual PA’s effect on peak $\overset{\cdot }{V}O_{2}$ remains equivocal [34]. Given that the training histories were comparable between sexes, it remains to be established whether this sex difference in the magnitude of training-related differences is attributable to physiological or methodological factors, or a combination thereof.

In the current study, boys had a significantly higher peak $\overset{\cdot }{V}O_{2}$ compared to girls (23.7%), even after allometrically scaling for body mass (21.1%). These findings are congruent with previous research in which boys of varying training statuses had a 12.8–22.5% higher peak $\overset{\cdot }{V}O_{2}$ whether expressed in absolute [12,13,14,18] or allometrically scaled [6,7,8,15,18] terms, compared to maturity matched girls. The mechanisms underpinning these sex differences in peak $\overset{\cdot }{V}O_{2}$ however remain to be fully elucidated [4,19]. Specifically, Rowland et al. [35] and Vinet et al. [36] found a higher SV_max_, quantified by Doppler echocardiography, in boys whereas in accord with the current study, Winsley et al. [18] reported no significant differences in SV_max_ or $\overset{\cdot }{Q}$_max_ when assessed using bioelectrical thoracic impedance. Furthermore, there was no difference in the SV response profile between any group in the present study. Such findings corroborate those of Obert et al. [16] and Nottin et al. [37] but contradict those of McNarry et al. [15]. These findings may reflect the lower training volume and history of the present participants compared to those in McNarry et al. [15] given the length of time needed to engender significant morphological adaptations within the myocardium [17]. Alternatively, they may also be attributable to a greater change in the peripheral vasculature, as opposed to central oxygen delivery, in response to training, as suggested by Obert et al. [16].

Extending the findings of Winsley et al. [18], the present study suggests that changes in oxygen extraction may be more important to the development of peak $\overset{\cdot }{V}O_{2}$ and the sexual dimorphism demonstrated. Indeed, McNarry et al. [23] reported sex differences in muscle deoxygenation kinetics, with pre-pubertal girls (age: 9.9 ± 0.6 years) demonstrating a greater rate of change in the [HHb] response compared to boys. The results of the current study corroborate these results and extend them across adolescence, with boys, irrespective of training, demonstrating a higher *c*/*d* and plateau when [HHb] was expressed against absolute $\overset{\cdot }{V}O_{2}$ and work rate. These sex differences may be related to a lower muscle oxidative capacity in girls, or a lower ability to redistribute blood to the metabolically active myocytes [38]. More specifically, research has reported that a greater proportion of blood flow in women is directed to the respiratory muscles [38]. Furthermore, women have a higher cost of breathing compared to men therefore potentially having less of a reserve to redistribute blood, and subsequently oxygen, to the peripheral vasculature [38], but further work needs to be carried out in children to confirm this hypothesis.

Despite sex differences in the [HHb] response when expressed against absolute work rate and $\overset{\cdot }{V}O_{2}$, when the [HHb] response was expressed against relative $\overset{\cdot }{V}O_{2}$ or work rate, the sex differences were ameliorated, suggesting that for the same relative sub-maximal work rate, boys and girls have a similar response pattern [22]. However, the normalisation of $\overset{\cdot }{V}O_{2}$ and work rate to peak values do not necessarily indicate boys and girls experience the same relative intensity of exercise. Indeed, the intensity of exercise is dependent upon the GET and critical power thresholds [2,3] and given the similarity in the GET between boys and girls, irrespective of training status, this study tentatively suggests that oxygen extraction capacity at sub-maximal intensities remains similar throughout maturation in boys and girls. Therefore, further research is required to determine the mechanistic basis for the sex differences in peak $\overset{\cdot }{V}O_{2}$.

Few studies have considered the effect of sex, or its interaction with training, on the sub-maximal parameters of aerobic fitness, with the exception of the GET for which evidence consistently reports that no sex differences are manifest when GET is normalised for peak $\overset{\cdot }{V}O_{2}$ [7,8,18]. The current findings that the GET was not affected by training status agree with the findings of McNarry et al. [15] who reported that the relative GET was similar during lower body exercise irrespective of training status. However, in contrast to McNarry et al. [15], the MRT of the current trained participants was significantly slower than their untrained counterparts. This finding also contradicts most evidence available regarding the influence of training on the dynamic $\overset{\cdot }{V}O_{2}$ response to constant work rate exercise [23,39]. However, the $\overset{\cdot }{V}O_{2}$ response to the incremental ramp and constant work rate exercise, and its determinants, could be dissociated. The lower gain in the trained youth is similarly counter-intuitive, with previous studies in adults suggesting that the proportion of type I fibres, and possibly fitness, are associated with a greater gain [22,23,28]. It is therefore interesting to note the greater gain reported in the boys in the current study irrespective of training status. However, given the discrepancy in the present training and testing modality, the discrepant findings regarding the influence of training on the submaximal parameters of aerobic fitness may also be methodological, rather than physiological, but further research is needed to confirm this.

Whilst there are strengths associated with the present study, including a large sample of trained and untrained children and adolescents and the use of appropriate scaling techniques, there are some limitations that must be acknowledged. First, given the sex differences in body composition, more insight may have been gained by scaling by fat-free mass or lean body mass. Further, all trained children and adolescents were part of similar training regimes, namely football and hockey, precluding any inferences being drawn regarding the effect of different training types. Additionally, the interpretation of the [HHb] signal has specific methodological limitations, including the generalisability of response dynamics from a singular localised area to the whole muscle, and variations in adiposity between boys and girls [22]. Finally, not splitting sex and training groups by maturity precludes inferences as to whether the observed differences are consistent across maturity, or whether there are periods of divergence. 

## 5. Conclusions

The purpose of this study was to examine the mechanisms underpinning sexual dimorphism in peak $\overset{\cdot }{V}O_{2}$. In conclusion, boys had a greater peak $\overset{\cdot }{V}O_{2}$ than girls, irrespective of training status, which does not appear to be related to differences in oxygen delivery as SV_max_ and $\overset{\cdot }{Q}$_max_ were similar between sexes when appropriately normalised to body surface area. This study indicates that the sex and training differences in peak $\overset{\cdot }{V}O_{2}$ may rather be due to enhanced oxygen extraction at the working muscles. Future research should seek to investigate sex differences across each maturational stage and investigate the potential mechanisms underpinning peak $\overset{\cdot }{V}O_{2}$ development in youth. 

## Figures and Tables

**Table 1:** Descriptive characteristics.

	Trained (*n* = 108)		Untrained (*n* = 108)	
	Boys (*n* = 65)	Girls (*n* = 43)	Boys (*n* = 65)	Girls (*n* = 43)
Age (years)	14.2 ± 1.9	14.4 ± 1.7	14.7 ± 1.7	14.6 ± 1.8
Height (m)	1.64 ± 0.15	1.62 ± 0.09	1.68 ± 0.11	1.60 ± 0.09
Weight (kg)	52.4 ± 12.9	53.2 ± 9.3	60.5 ± 13.0	53.6 ± 10.7
BMI (kg·m^−2^)	19.3 ± 2.4	20.2 ± 2.2	21.2 ± 3.5	20.5 ± 2.9
Maturity Offset (years)	0.21 ± 1.78	0.54 ± 1.22	0.83 ± 1.61	0.77 ± 1.46
MVPA (mins·day^−1^)	58.6 ± 24.4	48.2 ± 19.0 *	50.9 ± 19.0	46.6 ± 16.8 *
SED (mins·day^−1^)	554.3 ± 88.6	541.3 ± 74.4	528.8 ± 107.1	547.5 ± 131.2

All values are presented as mean ± SD, BMI = Body Mass Index, MVPA = Moderate-to-Vigorous Physical Activity, SED = Sedentary Time. * Indicates a significant difference compared to boys.

**Table 2:** Pulmonary gas exchange and hemodynamic responses to incremental ramp exercise according to training status and sex.

	Trained		Untrained	
	Boys	Girls	Boys	Girls
Oxygen Uptake and Power Variables				
Peak $\overset{\cdot }{V}O_{2}$ (L min^−1^)	2.64 ± 0.70 *^,#^	2.11 ± 0.40 *	2.66 ± 0.67 ^#^	1.93 ± 0.50
Allometrically Scaled Peak $\overset{\cdot }{V}O_{2}$ (mL·kg^−b^·min^−1^)	192.2 ± 28.6 *^,#^	152.5 ± 23.5 *	174.6 ± 33.8 ^#^	140.5 ± 34.3
GET (L min^−1^)	1.55 ± 0.49 ^#^	1.25 ± 0.26	1.55 ± 0.36 ^#^	1.20 ± 0.31
Relative GET (% Peak $\overset{\cdot }{V}O_{2}$)	59.4 ± 10.3	60.2 ± 10.8	59.6 ± 11.2	63.6 ± 12.7
MRT (s)	32.9 ± 16.2 *^,#^	30.7 ± 16.8 *	29.5 ± 24.4 ^#^	20.0 ± 13.7
Gain (mL·min^−1^·W^−1^)	8.8 ± 2.1 ^#^	7.6 ± 1.5	9.6 ± 2.5 ^#^	7.9 ± 2.0
Peak Power (W)	219 ± 66 *^,#^	205 ± 43 *	211 ± 51 ^#^	177 ± 41
Relative Peak Power (W∙Kg^−1^)	4.2 ± 1.3	3.9 ± 0.8	3.5 ± 0.9	3.3 ± 0.8
Cardiac Variables				
HR_max_ (beats·min^−1^)	199 ± 11	194 ± 11	195 ± 12	192 ± 13
SV_max_ (mL)	134.5 ± 53.9 *^,#^	130.1 ± 35.3 *	131.2 ± 42.1 ^#^	108.4 ± 29.3
Allometrically Scaled SV_max_ (mL·m^−b^)	74.9 ± 26.9	72.6 ± 22.2	68.6 ± 24.7	63.9 ± 17.3
$\overset{\cdot }{Q}$_max_ (L min^−1^)	21.9 ± 8.5 *	20.5 ± 6.6 *	18.5 ± 6.2	14.3 ± 5.1
Allometrically Scaled $\overset{\cdot }{Q}$_max_ (L m^−b^·min^−1^)	12.5 ± 4.0	12.0 ± 4.2	12.5 ± 3.8	11.5 ± 3.1
a-$\bar{v}O_{2}$ diff (mL·dL^−1^)	14.6 ± 6.7 ^#^	10.3 ± 2.4	13.6 ± 4.9 ^#^	10.8 ± 2.6

GET = Gas Exchange Threshold, MRT = Mean Response Time, HR_max_ = Maximum Heart Rate, SV_max_ = Maximum Stroke Volume, $\overset{\cdot }{Q}$_max_ = Maximum Cardiac Output, a-$\bar{v}$O_2_ diff = arteriovenous difference. All values presented as mean ± SD, * Indicates a significant difference between training groups within a sex. ^#^ Indicates a significant difference between sexes within a training group.

**Table 3:** Muscle deoxygenation variables from the incremental ramp exercise according to training status and sex.

		Trained		Untrained	
		Boys	Girls	Boys	Girls
[HHb] vs. absolute $\overset{\cdot }{V}O_{2}$	a (%)	95.9 ± 10.6	97.9 ± 9.5	96.2 ± 8.8	95.6 ± 8.7
	d	2.80 ± 1.50 *	2.67 ± 1.55 *	3.39 ± 2.93	3.95 ± 3.73
	*c*/*d*	1.58 ± 0.49 *^,#^	1.39 ± 0.45 *	1.50 ± 0.52 ^#^	0.89 ± 0.40
	Plateau	2.05 ± 0.44 *^,#^	1.68 ± 0.56 *	1.87 ± 0.61 ^#^	1.39 ± 0.25
[HHb] vs. relative $\overset{\cdot }{V}O_{2}$ (% Peak $\overset{\cdot }{V}O_{2}$)	a (%)	95.9 ± 10.6	97.9 ± 29.5	96.2 ± 30.8	95.6 ± 8.7
	d	0.07 ± 0.05	0.05 ± 0.03	0.10 ± 0.09	0.04 ± 0.02
	*c*/*d*	63.4 ± 15.5	58.4 ± 9.4	53.2 ± 15.8	50.1 ± 19.6
	Plateau	78.1 ± 12.6	71.4 ± 15.5	70.6 ± 16.8	76.1 ± 14.5
[HHb] vs. Watts (W)	a (%)	98.5 ± 36.7	95.4 ± 30.2	94.0 ± 12.9	93.8 ± 16.1
	d	0.02 ± 0.02 *	0.02 ± 0.01 *	0.08 ± 0.07	0.03 ± 0.01
	*c*/*d*	100 ± 29 *^,#^	91 ± 25 *	82 ± 29 ^#^	81 ± 19
	Plateau	162 ± 64 *^,#^	151 ± 63 *	136 ± 45 ^#^	128 ± 31
[HHb] vs. Watts (% Peak Power)	a (%)	98.5 ± 36.7	95.4 ± 30.2	94.0 ± 12.9	93.8 ± 16.1
	d	0.03 ± 0.02	0.04 ± 0.02	0.02 ± 0.01	0.04 ± 0.03
	*c*/*d*	49.8 ± 17.2	47.8 ± 16.8	45.1 ± 15.0	40.6 ± 18.0
	Plateau	80.8 ± 16.4 *	70.6 ± 19.7 *	68.9 ± 21.4	67.5 ± 21.4

[HHb] = concentration of haemoglobin, a = sigmoidal amplitude, d = sigmoidal slope, *c*/*d* = Value at the mid-point of the sigmoidal response, Plateau = Value at the lower 95% confidence interval of the amplitude. All values presented as mean ± SD, * Indicates a significant difference between trained and untrained within a sex. ^#^ Indicates a significant sex difference within the same training group.

## Data Availability

The data presented in this study are available on request from the corresponding author. The data are not publicly available due to ethical considerations when working with children and young people.
